# Managing a Locally Advanced Cervix Cancer Patient With COVID-19: Lessons Learned

**DOI:** 10.7759/cureus.10138

**Published:** 2020-08-30

**Authors:** David D'Souza, Vikram M Velker, Lucas Mendez, Pencilla Lang, Akira Sugimoto

**Affiliations:** 1 Radiation Oncology, London Regional Cancer Program, Western University, London, CAN; 2 Radiation Oncology, London Regional Cancer Program, Western University, London, CAN; 3 Gynecologic Oncology, London Regional Cancer Program, Western University, London, CAN

**Keywords:** cervix cancer, covid-19, radiotherapy, brachytherapy, chemotherapy

## Abstract

We present the case of a woman diagnosed with coronavirus disease 2019 (COVID-19) while undergoing chemoradiation for locally advanced cervix cancer. This diagnosis had implications for the treatment of her cancer, and a number of important decisions had to be made. We present the issues that arose and how her oncologic care was managed.

## Introduction

Primary radiotherapy provides definitive treatment for locally advanced cervix cancer and remains a priority that should not be deferred because of the pandemic [1]. Inferior outcomes are associated with prolonged overall treatment times of >56 days [2,3]. While much has been published on how resources should be used during the pandemic [4], there is little on how to manage a patient diagnosed with coronavirus disease 2019 (COVID-19) while receiving treatment. Given the implications to prolonging overall time, the need to deliver treatment presents challenges while ensuring the health of the patient and the safety of health care providers.

## Case presentation

**Figure 1:**
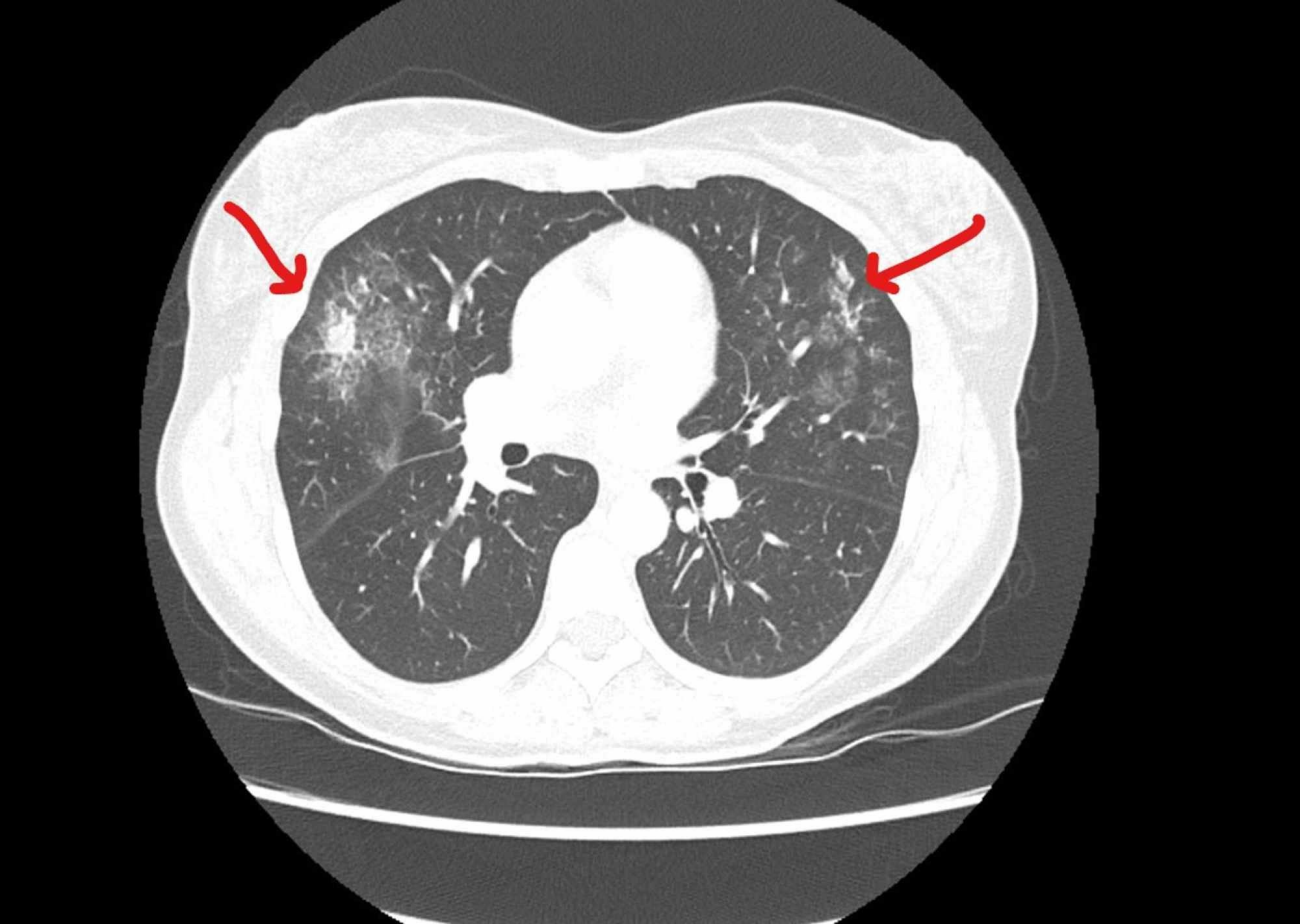
Axial CT slice showing bilateral pulmonary infiltrates

A 47-year-old woman presented in March 2020 with a FIGO (International Federation of Gynecology and Obstetrics) IIIB squamous cell cancer of the cervix, 6 cm primary with bilateral parametrial extension, and right-sided hydroureter. The recommended treatment plan was radiotherapy (external beam and brachytherapy) with concurrent weekly cisplatin chemotherapy. Due to pelvic pain, she was started on hydromorphone but her pain continued to increase, with an intensity of 10/10 when starting radiation and chemotherapy. After her second fraction, she required inpatient unit admission for pain crisis. On the fifth day of hospitalization, she was noted on routine vitals to have a temperature of 38.5°C. A febrile workup was initiated, including a chest X-ray, which showed an opacity in the right upper lobe of the lung. Her oxygen saturation was 97% on room air. A provisional diagnosis of pneumonia was made, and a nasal swab was taken for COVID-19. CT of the chest was performed showing bilateral upper lobe ground-glass infiltrates (Figure 1). Twelve hours later, results of PCR were positive for COVID-19. On review of systems, she had no other symptoms and no known contacts or travel history. 

## Discussion

In order to make oncological decisions on how to proceed with this case in light of COVID-19 infection, multidisciplinary input and the opinion of experts in gynecologic oncology were sought and reviewed with the infection control service.

With the knowledge that delays in delivering radiation, either initiation or interruption, leads to a lower likelihood of tumor control, we prioritized finding a way to deliver radiation. It was felt that external beam radiotherapy should continue unless her medical condition deteriorated. The following day, her temperature was 37.8°C, but she did not display any respiratory symptoms; therefore, a decision was made to continue with daily external beam treatments. If a patient tests positive for COVID-19, an evaluation of the impact of the infection on the patient should be made. Where possible, proceeding or continuing with treatment should be strived for. Modifications or delays in treatment should be evaluated based on clinical signs and symptoms with careful monitoring. Ultimately, a decision requires balancing the risks of continuing cancer treatment against that of COVID-related complications to the patient and potential exposure to other patients and staff.

Our patient was transferred to a dedicated COVID-19 floor in the hospital and scheduled as the last treatment of the day. This allowed her to be transferred from the inpatient unit to the treatment machine at a less crowded time. Standard personal protective equipment (PPE) (mask, gloves, and gown) was required by all involved in her care including radiation therapists. By being treated at the day’s end, a full cleaning could be performed without delaying the workflow on the treatment unit. Contact tracing was provided for staff in contact with the patient in the preceding 72 hours prior to becoming symptomatic. At the time, COVID testing was not available for asymptomatic staff and they were placed in self-isolation for 14 days. No staff became symptomatic. It was helpful to seek the guidance provided by the infection control service on appropriate measures and when they could be lifted.

The elevated temperature resolved within 24 hours with no further symptoms. Pain control was also optimized in the hospital, improving due to a combination of opioid rotation and likely tumor response to radiation. She was cleared to be discharged to social isolation on the 11th day in the hospital. Arrangements were made to continue radiation as an outpatient, with a mask being provided to the patient. Her treatment remained at the end of the day, and she was given a number to call when she arrived in the parking lot so that she could be taken directly to the treatment unit. The infectious diseases service provided guidance that 14 days after testing positive if the patient remained asymptomatic, then special precautions no longer were needed. As a result, the patient was not asked to wear a mask after 14 days and her treatment schedule was no longer restricted. Since that time, our understanding and policies have changed that all patients wear a mask irrespective of their COVID status. The risk of reinfection or being infectious beyond 14 days with aerosol-generating medical procedures is unclear. Prior engagement of relevant disciplines (e.g. anesthesia) helped ensure that brachytherapy could proceed. Where resources exist, routine pre-brachytherapy COVID-19 testing may reduce risk to staff, though donning of proper PPE and precautions should be taken regardless.

The opinion of experts was solicited on the continued administration of cisplatin. While it was agreed to hold giving her an infusion after a diagnosis was made, there was uncertainty if and when it would be safe to resume. It was suggested to obtain a negative repeat COVID-19 swab, but it was unclear how this should be interpreted. In view of her relatively young age and otherwise excellent health, there was reluctance to forgo the potential improvement in survival without clear data that chemosensitization was contraindicated. In the absence of any established parameters to provide guidance, a decision was made to resume cisplatin based on clinical judgment and shared decision making with the patient, taking into account her status and functioning. After being given on day 1, cisplatin was held for days 8 and 15. After it was clear that the infection had resolved, cisplatin was resumed for days 22, 29, and 36, with no noted complications.

With bulky disease causing hydronephrosis, we anticipated using a hybrid tandem/ring applicator with interstitial needles. Anesthesia requested a repeat COVID-19 swab test that was performed even though well beyond 14 days of remaining symptom-free. This was requested in case spinal anesthesia could not be administered. Testing was negative. The first brachytherapy procedure was performed under spinal anesthesia with two fractions given over 24 hours. On presentation for the second brachytherapy procedure, the patient developed a fever (38.3°C). The febrile workup included repeat COVID-19 testing that came back negative. The source of infection was identified as the urinary tract related to her nephrostomy tube. She proceeded with brachytherapy as scheduled with spinal anesthesia.

With the last fraction of brachytherapy, radiation treatment was completed in 46 days with four cycles of cisplatin chemotherapy given. The patient was discharged in stable condition with no apparent acute complications or readmissions in the three weeks elapsed since treatment completion.

This patient was the first COVID-positive cancer patient on treatment within our community early in the pandemic where the trajectory and impact on hospital resources were unknown. Strong communication and pre-existing relationships in the health care team prior to the pandemic were essential. This allowed us to make decisions on how to proceed expediently with the trust of all involved in providing care.

## Conclusions

Cervix cancer is a significant cause of cancer mortality worldwide, particularly in young women. Primary radiotherapy provides definitive treatment, and treatment should remain a priority during the COVID-19 pandemic. It is important to strike a balance between managing the infection and timely oncologic care without delays. Multidisciplinary engagement of involved health care workers is important to ensure adequate communication and ensuring the safety of all providing care.
